# Endogenous dynorphin protects against neurotoxin-elicited nigrostriatal dopaminergic neuron damage and motor deficits in mice

**DOI:** 10.1186/1742-2094-9-124

**Published:** 2012-06-13

**Authors:** Qingshan Wang, Eun-Joo Shin, Xuan-Khanh Thi Nguyen, Quan Li, Jae-Hyung Bach, Guoying Bing, Won-Ki Kim, Hyoung-Chun Kim, Jau-Shyong Hong

**Affiliations:** 1 Neuropharmacology Section, Laboratory of Toxicology and Pharmacology, National Institute of Environmental Health Sciences, Research Triangle Park, NC, 27709, USA; 2 Neuropsychopharmacology and Toxicology Program, College of PharmacyKangwon National University, Chunchon, 200-701, South Korea; 3 Department of Anatomy and Neurobiology, College of Medicine, University of Kentucky, Lexington, KY, 40536, USA; 4 Department of Neuroscience, College of Medicine, Korea University, Seoul, 136-705, South Korea

**Keywords:** Parkinson’s disease, 1-methyl-4-phenyl-1,2,3,6-tetrahydropyridine, methamphetamine, neuroinflammation, microglia, dynorphin, prodynorphin-deficient mice, nigrostriatal dopaminergic toxicity, behavioral deficit.

## Abstract

**Background:**

The striato-nigral projecting pathway contains the highest concentrations of dynorphin in the brain. The functional role of this opioid peptide in the regulation of mesencephalic dopaminergic (DAergic) neurons is not clear. We reported previously that exogenous dynorphin exerts potent neuroprotective effects against inflammation-induced dopaminergic neurodegeneration *in vitro*. The present study was performed to investigate whether endogenous dynorphin has neuroprotective roles *in vivo*.

**Methods:**

1-Methyl-4-phenyl-1,2,3,6-tetrahydropyridine (MPTP) and methamphetamine (MA), two commonly used neurotoxins in rodent models of Parkinson’s disease, were administered to wild-type (Dyn^+/+^) and prodynorphin-deficient mice (Dyn^−/−^). We examined dopaminergic neurotoxicity by using an automated video tracking system, HPLC, immunocytochemistry, and reverse transcription and polymerase chain reaction (RT-PCR).

**Results:**

Treatment with MPTP resulted in behavioral impairments in both strains. However, these impairments were more pronounced in Dyn^-l-^ than in Dyn^+/+^. Dyn^−/−^ showed more severe MPTP-induced dopaminergic neuronal loss in the substantia nigra and striatum than Dyn^+/+^. Similarly, the levels of dopamine and its metabolites in the striatum were depleted to a greater extent in Dyn^−/−^ than in Dyn^+/+^. Additional mechanistic studies revealed that MPTP treatment caused a higher degree of microglial activation and M1 phenotype differentiation in Dyn^−/−^ than in Dyn^+/+^. Consistent with these observations, prodynorphin deficiency also exacerbated neurotoxic effects induced by MA, although this effect was less pronounced than that of MPTP.

**Conclusions:**

The *in vivo* results presented here extend our previous *in vitro* findings and further indicate that endogenous dynorphin plays a critical role in protecting dopaminergic neurons through its anti-inflammatory effects.

## Background

Dynorphin is an endogenous opioid peptide system that is widely distributed in various tissues. In the central nervous system (CNS), the neurons projecting from the striatum to the substantia nigra (SN) contain high levels of dynorphin, which co-exists with substance P. The roles of these two peptides in motor function regulation in the striatonigral pathway have been studied extensively over the past decade [1]. Dynorphin and substance P have been shown to regulate nigrostriatal dopamine (DA) release, motor behavior, and learning in a reciprocal manner [2-5]. Conversely, the nigrostriatal DAergic systems also serve to up-regulate dynorphin gene expression by interacting with D1 receptor and subsequently phosphorylating the cAMP response elements (CRE) within the prodynorphin gene promoter [6,7]. These earlier reports indicate an intimate reciprocal relationship between dynorphin and substance P in regulating DAergic neurons in the basal ganglia and further suggest possible roles of these two peptides in movement disorders, such as Parkinson’s disease (PD).

Numerous studies have suggested the involvement of dynorphin in the pathogenesis of PD and as a mechanism of action of l-DOPA (l-3,4-dihydroxyphenylalanine) therapy. Reduced mRNA levels of prodynorphin were observed in the SN in postmortem brain specimens of PD patients by quantitative PCR [8]. Similar findings were also reported in animal models of PD [9]. For example, in the 6-hydroxydopamine (6-OHDA)-induced rat model of PD, mRNA levels of dynorphin were decreased in the striatum as compared with controls. Furthermore, the observed decreases in dynorphin mRNA levels in these 6-OHDA rats returned to near control levels after administration of levodopa (l-DOPA) or SKF38393 (D1 DAergic receptor agonist) [9]. These findings further suggest the possible involvement of dynorphin in the pathogenesis of PD. Furthermore, excessive expression of opioid neuropeptides, such as dynorphin and enkephalin, was closely associated with l-DOPA-induced dyskinesias in DA-denervated animal PD models [10]. In contrast, the non-selective opioid antagonist naloxone attenuated l-DOPA-induced involuntary movements in 1-methyl-4-phenyl-1,2,3,6-tetrahydropyridine (MPTP)-treated common marmosets [11].

Although previous studies revealed a critical role of dynorphin in the regulation of DAergic neuronal functions and involvement in the pathogenesis of PD, little is known regarding its function in regulating DAergic neuron survival. Our previous studies showed potent neuroprotective effects of dynorphin in inflammation-induced DAergic neuron toxicity. In mesencephalic neuron-glia cultures, we demonstrated that addition of ultra-low concentrations (10^-13^-10^-15^ M) of dynorphin protected DAergic neurons against lipopolysaccharide (LPS)-induced neurotoxicity [12]. Both dynorphin A (1–17) and the receptor binding ineffective [des-Tyr^1^ dynorphin A (2–17) exerted equal potency, indicating that the neuroprotective effect of dynorphin is independent of κ-opioid receptors. Further studies showed that dynorphin protected DAergic neurons against substance P-induced reduction of DA uptake and cell body loss [13]. Detailed mechanistic studies revealed that anti-inflammatory effects, such as the inhibition of NADPH oxidase-generated superoxide and proinflammatory factors from microglia, mediated the neuroprotective effects of dynorphin [14].

The cell culture studies described above provided strong evidence indicating a critical neuroprotective role of dynorphin; however, there have been no reports describing the functional relevance of endogenous dynorphin in the pathogenesis of PD. For this purpose, prodynorphin-deficient mice (Dyn^−/−^) and wild-type mice (Dyn^+/+^) were treated with MPTP or methamphetamine (MA), two commonly used neurotoxins for rodent PD models. Here, we report that endogenous dynorphin deficiency exacerbates MPTP- or MA-induced motor deficits, loss of nigrostriatal DAergic neurons, and reduction of striatal DA level. The mechanism might be related to the higher degrees of microglial activation and M1 phenotype differentiation in Dyn^−/−^. Thus, our results suggest that endogenous prodynorphin knockout adversely affects the progression of PD, supporting the potential neuroprotective role of endogenous dynorphin in PD.

## Methods

### Reagents

We purchased MPTP, MA, DA, 3, 4-dihydroxyphenylacetic acid (DOPAC), and homovanillic acid (HVA) from Sigma Chemical Co. (St. Louis, MO, USA). Antibodies to tyrosine hydroxylase and Iba-1 were obtained from Millipore (Temecula, CA, USA) and from Wako Pure Chemical Industries, Ltd. (Osaka, Japan), respectively. RNeasy Mini kits were purchased from Qiagen (Valencia, CA, USA). The primers for RT-PCR, including Arginase1, CD206, CD16, CD32, and CD86, were obtained from Bioneer Corporation (Daejeon, South Korea). All other reagents were of analytical or high-performance liquid chromatography (HPLC) grade.

### Animals

All animals were treated in accordance with the National Institutes of Health (NIH) Guide for the Humane Care and Use of Laboratory Animals (NIH Publication No. 85–23, 1985; http://www.dels.nas.edu/ila). The present study was performed in accordance with the Institute for Laboratory Research (ILAR) guidelines for the care and use of laboratory animals. Mice were maintained under a 12-h light:12-h dark cycle and fed *ad libitum*. The Dyn^−/−^ mice were originally obtained by targeted deletion of the coding exons of the prodynorphin gene [15]. We used this animal model in our previous studies [16,17]. The Dyn^−/−^ strain used in the present study was backcrossed at least nine times to the C57BL/6 background. Prior to weaning, tail specimens were collected from each animal, and DNA was extracted to confirm the presence of the prodynorphin gene locus by polymerase chain reaction (PCR) using primer pairs specific for each genotype [15-17]. Primers to detect WT alleles at the prodynorphin gene locus were 5′-CAGGACCTGGTGCCGCCCTCAGAG-3′ and 5′-CGCTTCTGGTTGTCCCACTTCAGC-3′; primers specific for the deletion were 5′-ATCCAGGAAACCAGCAGCGGCTAT-3′ and 5′-ATTCAGACACATCCCACATAAGGACA-3′. The products were amplified in a GeneAmp PCR System 9700 (Applied Biosystems, Foster City, CA, USA) using the following PCR parameters: an initial denaturation at 94°C for 5 min, and then 30 cycles of 94°C for 30 s, 65°C for 30 s, 72°C for 30 s, and 72°C for 5 min, followed by electrophoresis on 1% agarose gels with ethidium bromide and photography under ultraviolet (UV) light.

Because young mice (that is 8- to 10-week old animals) are known to be less sensitive to behavioral impairments induced by dopaminergic neurotoxins (for example, MPTP) than adult mice [18] and show spontaneous behavioral recovery within several days after subchronic treatment with MPTP [18,19], we employed 6-month-old mice with a Dyn^+/+^ or Dyn^−/−^ genotype to better understand behavioral impairments induced by both dopaminergic toxins. Under our experimental condition, spontaneous behavioral recovery was not observed until at least 7 days after the final MPTP or MA treatment (see Additional file 1 and Additional file 2: Figure S1).

### Drugs treatment

Mice received four injections of MA (5 or 7 mg/kg, i.p.) at 2-h intervals. MPTP (15 or 20 mg/kg, i.p.) was injected once daily for 7 consecutive days. The dose of MPTP or MA was determined based on previous studies [20-24] and our pilot study [25]. The mice were sacrificed 1, 3, and 7 days after the final MA or MPTP treatment.

### Locomotor activity

Locomotor activities were measured for 30 min using an automated video-tracking system (Noldus Information Technology, Wagenin, The Netherlands) at 7 days after the final injection of MPTP or MA. Four test boxes (40 × 40 × 40 cm) were operated simultaneously by an IBM computer. Animals were studied individually during locomotion in each test box. Animals were allowed to acclimatize to the test box for 5 min before starting the experiment. A printout for each session showed the pattern of ambulatory movements in the test box. The distances traveled in centimeters by the animals during horizontal locomotor activity were analyzed [26].

### Rota-rod test

Rota-rod test was performed at 7 days after the final injection of MPTP or MA. The apparatus (model 7650; Ugo Basile, Comerio, Varese, Italy) consisted of a base platform and a rotating rod with a non-slip surface. The rod was placed at a height of 15 cm above the base. The rod, 30 cm in length, was divided into equal sections by six opaque disks so that the animals would not be distracted by one another. To assess motor performance, the mice were first trained on the apparatus for 2 min at a constant rate of 4 rpm. The test was performed 30 min after training and an accelerating paradigm was applied, starting from a rate of 4 rpm to maximal speed of 40 rpm. The rotation speed was then kept constant at 40 rpm. The latency to fall was measured with a maximal cutoff time of 300 s [27].

### Measurement of dopamine (DA), 3,4-dihydroxyphenylacetic acid (DOPAC), and homovanillic acid (HVA) level

Mice were killed by cervical dislocation and the brains were removed. The striatum was dissected, immediately frozen on dry ice, and stored at −70°C before assays were performed. Tissues were weighed, ultrasonicated in 10% perchloric acid, and centrifuged at 20,000 × *g* for 10 min. The levels of DA and its metabolites, DOPAC and HVA, in brain tissue extracts were determined by HPLC coupled with an electrochemical detector as described [28]. Supernatant aliquots (20 μL) were then injected into an HPLC equipped with a 3 μm C18 column. The mobile phase was comprised of 26 mL of acetonitrile, 21 mL of tetrahydrofuran, and 960 mL of 0.15 M monochloroacetic acid (pH 3.0) containing 50 mg/L of EDTA and 200 mg/mL of sodium octyl sulfate. The amounts of DA, DOPAC, and HVA were determined by comparison of peak areas of tissue samples with authentic standards, and were expressed in ng/g of wet tissue.

### Reverse transcription and polymerase chain reaction (RT-PCR)

Total RNA from the striatum was isolated using an RNeasy Mini kit (Qiagen). Reverse transcription was performed by incubation for 1 h at 37°C in reaction mixtures containing AMV transcriptase and random oligonucleotide primers. PCR amplification was performed for 35 cycles of denaturation at 94°C for 1 min, annealing at 60°C for 2 min, and extension at 72°C for 1 min. Primer sequences [29] for PCR amplification are listed in Table 1. PCR products were separated on 2% agarose gels containing ethidium bromide. Quantitative analysis of RNA was performed using PhotoCaptMw computer software (Vilber Lourmat, Marne-la-Vallée, France).

**Table 1:** Gene primer sequences for RT-PCR analysis

**Gene**	**Forward primer (5'-3')**	**Reverse primer (5'-3')**
Arginase 1	GAACACGGCAGTGGCT TTAAC	TGCTTAGCTCTGTCTGC TTTGC
CD206	TCTTTGCCTTTCCCAGTC TCC	TGACACCCAGCGGAAT TTC
CD16	TTTGGACACCCAGATGT TTCAG	GTCTTCCTTGAGCACCT GGATC
CD32	AATCCTGCCGTTCCTAC TGATC	GTGTCACCGTGTCTTCC TTGAG
CD86	TTGTGTGTGTTCTGGAAA CGGAG	AACTTAGAGGCTGTGTT GCTGGG
GAPDH	ACCACAGTCCATGCCAT CAC	TCCACCACCCTGTTGCT GTA

### Western blot analysis

Western blotting analysis was performed as described previously [26]. Tissues were homogenized in lysis buffer containing a 200 mM Tris–HCl (pH 6.8), 10% SDS, 5 mM ethylene glycol tetraacetic acid (EGTA), 5 mM ethylenediaminetetraacetic acid (EDTA), 10% glycerol, and protease inhibitor cocktail (Sigma). Lysates were centrifuged at 13,000 × *g* for 30 min and the supernatant fractions were used for Western blotting analysis. Proteins (20 μg/lane) were separated by 10% sodium dodecyl sulfate-polyacrylamide gel electrophoresis (SDS-PAGE) and transferred onto polyvinylidene fluoride (PVDF) membranes, and the resulting blots were blocked in phosphate buffered saline (PBS) containing 3% skim milk for 30 min. Each blot was incubated overnight at 4°C with the primary antibody against β-actin (1:50,000; Sigma), or TH (1:5,000; Chemicon, Temecula, MA, USA). After washing in PBS, membranes were incubated with HRP-conjugated secondary anti-rabbit IgG (1:5,000; GE Healthcare, Arlington Heights, IL, USA), or anti-mouse IgG (1:5000; Sigma) for 2 h. Subsequent visualization was performed using the enhanced chemiluminescence system (ECL plus®; GE Healthcare). Relative band intensities were quantified by PhotoCaptMw (version 10.01 for Windows; Vilber Lourmat).

### Immunohistochemistry

For immunocytochemical analysis [30], mice were perfused transcardially with 50 mL of ice-cold PBS (10 mL/10 g body weight) followed by 4% paraformaldehyde (20 mL/10 g body weight). Brains were removed and stored in 4% paraformaldehyde overnight. Sections were blocked with PBS containing 0.3% hydrogen peroxide for 30 min and then incubated in PBS containing 0.4% Triton X-100 and 1% normal serum for 20 min. After a 24-h incubation with primary antibody against TH (1:500; Chemicon) or Iba-1 (1:500; Wako), sections were incubated with the biotinylated secondary antibody (1:1000; Vector Laboratories, Burlingame, CA, USA) for 1 h. The sections were then immersed in a solution containing avidin-biotin peroxidase complex (Vector Laboratories) for 1 h, and 3,3'-diaminobenzidine was used as the chromogen. Digital images were acquired under an Olympus microscope (BX51; Olympus, Tokyo, Japan) using an attached digital microscope camera (DP72; Olympus) and an IBM PC.

The striatal densities of Iba-1 and TH immunoreactivity were measured using ImageJ version 1.44 software (National Institutes of Health, Bethesda, MD, USA) as described previously [31,32]. Briefly, the entire striatal region from each section was selected as the region of interest (ROI). Threshold values for hue (0–100), saturation (0–255), and brightness (175–255 for TH; 150–205 for Iba-1) were set in the ‘Adjust Color Threshold’ dialog box, and then the mean density was measured. Quantification was performed from four adjacent brain sections, spaced 120 μm apart, and was subsequently averaged for each animal.

### Stereological analysis

The total numbers of TH-immunoreactive neurons and activated microglia were estimated using the computerized optical fractionator method (Stereo Investigator ver. 7.5; MBF Bioscience, Microbrightfield, Inc., Williston, VT, USA) as described previously [33,34]. Briefly, a 5× objective lens was used to define the contours around the entire region of interest, and a 100× lens was used for assessment of TH- and Iba-1-immunoreactive cells following a systematically random sampling scheme. Serial sections covering the rostrocaudal extent of the SN were cut on a Microm HM440E microtome (cut thickness of 30 μm and final mounted thickness of 24 μm), and every sixth section was counted (a total of four sections) for systematic analysis of randomly placed counting frames (size of 50 × 50 μm) on a counting grid (size of 16000 μm^2^ area) and sampled using a 10-μm optical dissector with 2-μm upper and lower guard zones. A dopaminergic neuron was defined as a TH-immunoreactive cell body with a clearly visible TH-negative nucleus. The total numbers of TH- and Iba-1-immunoreactive cells from each animal were estimated using the serial section manager software. One series of each animal was analyzed for TH-IR and Iba-1-IR. The coefficient of error [35] was calculated to determine intra-animal variation and was less than 0.1 in all cases. For this stereological technology, the potential sources of bias mainly include the fixation and embedding processing protocol, immunostaining processing, and individual cell counting [36]. We took several precautions to minimize such influences: animal brains were fixed using the same protocol, tissues were cryosectioned using a cryostat microtome, the immunostaining steps were performed on all of the tissue sections simultaneously, and cell counting was conducted in a blind mode. Data were expressed as TH-positive cells [37,38].

### Statistics

**Figure 1:**
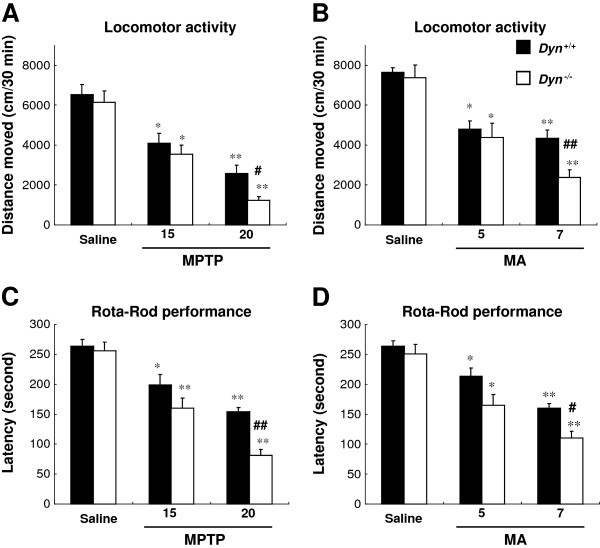
**Prodynorphin gene deficiency potentiates behavioral impairment induced by MPTP or MA.** Dyn^+/+^, prodynorphin gene wild-type mice; Dyn^−/−^, prodynorphin gene knockout mice. Dyn^+/+^ and Dyn^−/−^ received MPTP (15 or 20 mg/kg/day, i.p.) for 7 consecutive days or four MA injections (5 or 7 mg/kg, i.p.) at 2-h intervals. Behavioral changes were evaluated based on locomotor activity (**A and B**) and rota-rod tests (**C and D**). Each value is mean ± S.E.M of 12 mice. *P <0.01, **P < 0.001 vs. respective saline group, #*P* <0.01, ##*P* <0.001 vs. MPTP- or MA-treated Dyn^+/+^ (two-way ANOVA followed by *post-hoc* Tukey HSD test or pairwise comparison with paired *t*-test).

**Figure 2:**
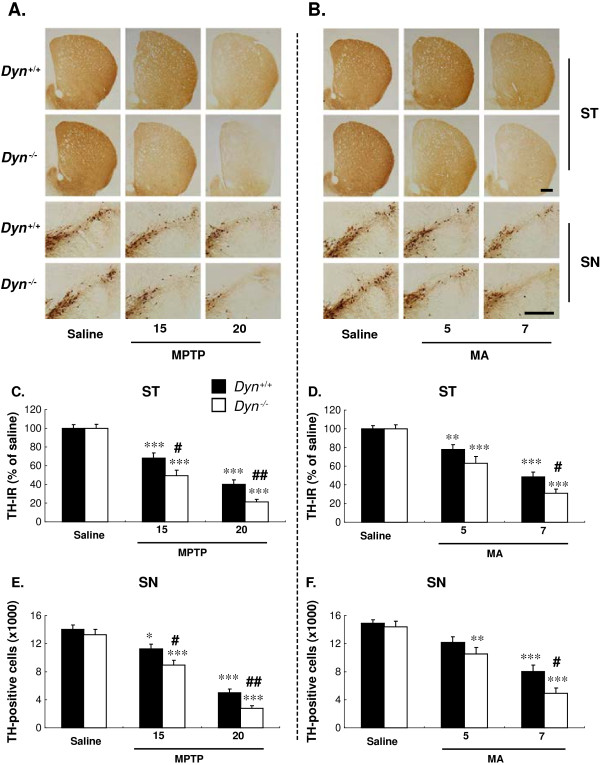
**Prodynorphin gene deficiency exacerbates striatonigral dopaminergic neurotoxicity induced by MPTP or MA.** Dyn^+/+^ and Dyn^−/−^ received MPTP (15 or 20 mg/kg, i.p./day × 7) or MA (5 or 7 mg/kg, i.p./2 h × 4) as described in Figure 1. Tyrosine hydroxylase (TH)-immunoreactive neurons were stained and counted 7 days after the last treatment with MPTP or MA by immunocytochemistry with an antibody against TH. Immunostaining of nigrostriatal TH-positive neurons treated with saline, MPTP (**A**), or MA (**B**) in Dyn^+/+^ and Dyn^−/−^. Immunostaining of striatal TH-positive fibers was quantified using ImageJ (**C, D**). Numbers of TH-positive neurons in the SN pars compacta were counted by stereological analyses (**E, F**). For more details, refer to ‘Methods’. ST = striatum. SN = substantia nigra*.* Each value is mean ± S.E.M of eight mice. *P <0.05, **P < 0.01, ***P < 0.001 vs. respective saline group, #*P* <0.05, ##*P* <0.01 vs. MPTP- or MA-treated Dyn^+/+^ (two-way ANOVA followed by *post-hoc* Tukey HSD test or pairwise comparison with paired *t*-test). Scale bar = 400 μm.

**Figure 3:**
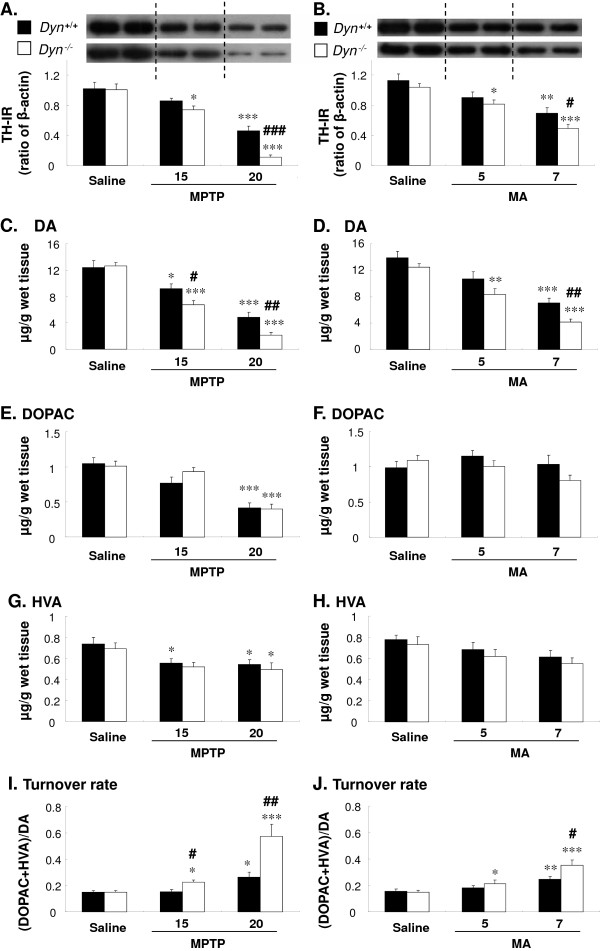
**Prodynorphin deficiency enhances MPTP- or MA-induced decreases in TH expression and DA level.** Dyn^+/+^ and Dyn^−/−^ received MPTP (15 or 20 mg/kg, i.p.) or MA (5 or 7 mg/kg, i.p.) as described in Figure 1. TH expression and DA and its metabolites were examined 7 days after the last treatment with MPTP or MA. Quantification of TH expression was performed as shown in **A** and **B**. The levels of dopamine (DA; **C, D**) and its metabolites, 3,4-hydroxyphenylacetic acid (DOPAC; **E, F**) and homovanillic acid (HVA; **G, H**), were determined by HPLC. The turnover rate was evaluated using the ratio of (DOPAC + HVA)/DA (**I, J**). Each value is mean ± S.E.M of eight mice. *P <0.05, **P < 0.01, ***P < 0.001 vs. respective saline group, #*P* <0.05, ##*P* <0.01, ###*P* <0.001 vs. MPTP- or MA-treated Dyn^+/+^ (two-way ANOVA followed by *post-hoc* Tukey HSD test or pairwise comparison with paired *t*-test).

**Figure 4:**
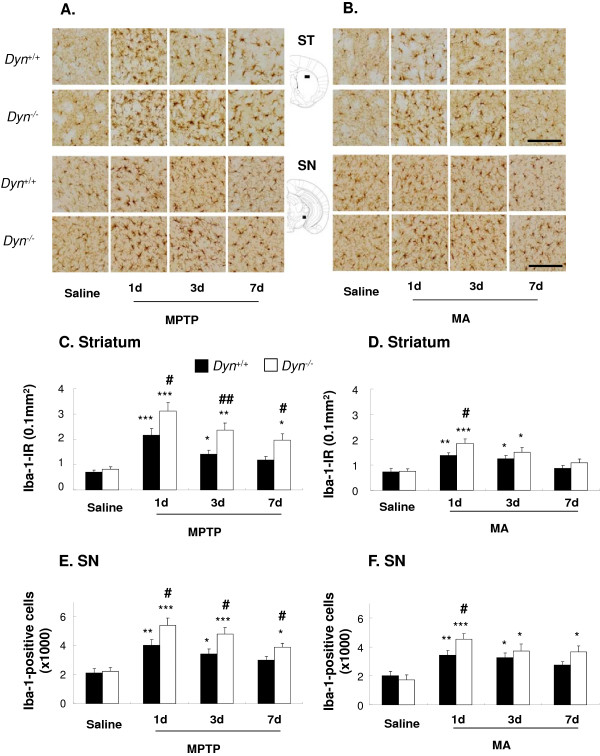
**Prodynorphin deficiency amplifies activation of microglia induced by MPTP or MA in the striatonigral areas.** Dyn^+/+^ and Dyn^−/−^ received MPTP (20 mg/kg, i.p./day × 7) or MA (7 mg/kg, i.p./2 h × 4) as described in Figure 1. Microglial activation induced by MPTP or MA was examined by immunocytochemistry with an antibody against Iba1. Striatal sections were collected at 0.62 mm anterior to bregma, while nigral sections were collected at 3.08 mm posterior to bregma according to the atlas of Franklin and Paxinos (2008). Representative photomicrographs of Iba1-immunoreactive cells in the striatum (ST) and substantia nigra (SN) of mice after the last treatment with saline, MPTP, or MA are shown (**A, B**). Immunostaining of striatal Iba1 immunoreactivity (Iba-1-IR) was quantified by immunoreactive density (**C, D**) and the number of Iba1-positive neurons in the SN pars compacta was counted by stereological analysis (**E, F**). For more details, refer to ‘Methods’. Each value is mean ± S.E.M of eight mice. *P <0.05, **P < 0.01, ***P < 0.001 vs. respective saline group, #*P* <0.05, ##*P* <0.01 vs. MPTP- or MA-treated Dyn^+/+^ (two-way ANOVA followed by *post-hoc* Tukey HSD test or pairwise comparison with paired *t*-test). Scale bar = 50 μm.

**Figure 5:**
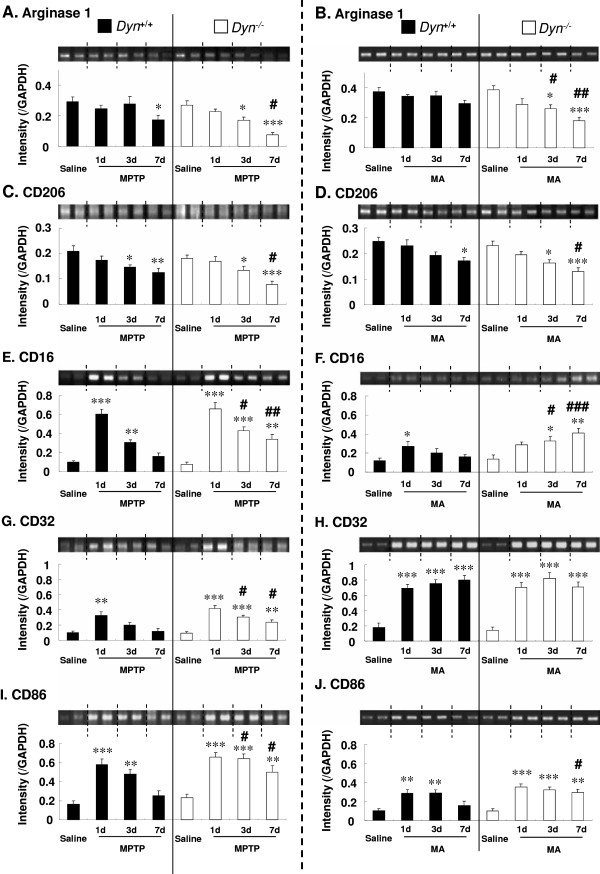
**Prodynorphin gene deficiency promotes microglial differentiation into M1 type after MPTP or MA administration.** Dyn^+/+^ and Dyn^−/−^ received MPTP (20 mg/kg, i.p./day × 7) or MA (7 mg/kg, i.p./2 h × 4) as described in Figure 1. Changes in mRNA levels associated with M2 (**A** to **D**) and M1 (**E** to **J**) phenotype markers induced by MPTP (**A, C, E, G, I**) or MA (**B, D, F, H, J**) in the striatum were evaluated by RT-PCR analysis. Each value is mean ± S.E.M of eight mice. *P <0.05, **P < 0.01, ***P < 0.001 vs. respective saline group, #*P* <0.05, ##*P* <0.01, ###*P* <0.001 vs. MPTP- or MA-treated Dyn^+/+^ (two-way ANOVA followed by *post-hoc* Tukey HSD test or pairwise comparison with paired *t*-test).

We followed statistical analyses outlined by Belin and Everitt [39] to understand the main effects and/or interactions with all appropriate values for F and P. Data were analyzed using two-way analysis of variance (ANOVA) with strains and doses (Figures 1,2, and 3) or with strains and time points (Figures 4 and 5) as between-subjects factors. When the main effect of strains, doses, or time points was significant, *post-hoc* Tukey’s HSD test (among groups in the same strain) or pairwise comparison using paired *t*-test (between two strains for each dose or each time point) was performed. A *P* value <0.05 was deemed statistically significant.

## Results

### Prodynorphin deficiency potentiates MPTP- or MA-induced behavioral impairment

To determine the roles of endogenous dynorphin in regulating motor function, locomotor activity and rota-rod performance [40,41] were examined in Dyn^+/+^ and Dyn^−/−^ after treatment with MPTP or MA, two widely used neurotoxins in rodent PD models. As shown in Figure 1, the locomotor activities in mice were significantly and dose-dependently decreased in response to MPTP (main effect of doses: *F*_2,66_ = 47.1, *P* < 0.001) or MA (main effect of doses: *F*_2,66_ = 37.8, *P* < 0.001), and the degree of impairment induced by MPTP appeared to be more pronounced than that by MA. Compared with Dyn^+/+^ mice, Dyn^−/−^ mice exhibited hypolocomotion after administration of MPTP (main effect of strains: *F*_1,66_ = 5.49, *P* < 0.05) or MA (main effect of strains: *F*_1,66_ = 4.92, *P* < 0.05) (Figure 1A and B). However, a significant interaction between doses and strains was not induced by MPTP (interaction of doses × strains: *F*_2,66_ = 1.15, *P* > 0.05) or MA (interaction of doses × strains: *F*_2,66_ = 1.85, *P* > 0.05). Changes in rota-rod performance were consistent with those in locomotor activity. The degree of impairment in rota-rod performance in Dyn^−/−^ mice was much more severe than that in Dyn^+/+^ mice (MPTP, main effect of doses: *F*_2,66_ = 57.5, *P* < 0.001 or strains: *F*_1,66_ = 13.4, *P* < 0.001; MA, main effect of doses: *F*_2,66_ = 43.7, *P* < 0.001 or strains: *F*_1,66_ = 12.0, *P* < 0.001) (Figure 1C and D). However, a significant interaction between doses and strains was not induced by MPTP (interaction of doses × strains: *F*_2,66_ = 2.91, *P* > 0.05) or MA (interaction of doses × strains: *F*_2,66_ = 1.33, *P* > 0.05). There were no significant differences in locomotor activity or rota-rod performance between saline-treated Dyn^+/+^ and Dyn^−/−^. These data suggested that endogenous dynorphin has a protective effect against the motor behavioral deficits induced by MPTP or MA.

### Prodynorphin deficiency exacerbates MPTP- or MA-induced DAergic neurotoxicity

To investigate whether endogenous dynorphin modulates DAergic neuronal survival *in vivo*, brain sections of SN and striatum from both Dyn^+/+^ and Dyn^−/−^ after MPTP or MA treatment were immunostained with an antibody against tyrosine hydroxylase (TH) (Figure 2A and B). Immunocytochemical analysis revealed a dose-dependent decrease in staining intensity of TH-positive bodies in the SNpc and fibers in the striatum after MPTP or MA treatment as compared with saline-injected controls. Quantification of TH-positive neurons and TH expression in SNpc (MPTP, main effect of doses: *F*_2,42_ = 120, *P* < 0.001; MA, main effect of doses: *F*_2,42_ = 51.8, *P* < 0.001) and striatum (MPTP, main effect of doses: *F*_2,42_ = 106, *P* < 0.001; MA, main effect of doses: *F*_2,42_ = 70.6, *P* < 0.001) (Figure 2C-F) confirmed the histological observations. Further analysis indicated a greater loss of dopaminergic neurons in Dyn^−/−^ mice than in Dyn^+/+^ mice (MPTP, main effect of strains: *F*_1,42_ = 12.5, *P* < 0.01 in the striatum and *F*_1,42_ = 11.7, *P* < 0.01 in the SNpc; MA, main effect of strains: *F*_1,42_ = 6.89, *P* < 0.05 in the striatum and *F*_1,42_ = 7.02, *P* < 0.05 in the SNpc) (Figure 2C-F). However, no significant interaction between doses and strains was induced by MPTP (interaction of doses × strains: *F*_2,42_ = 1.87, *P* > 0.05 in the striatum and *F*_2,42_ = 0.907, *P* > 0.05 in the SNpc) or MA (interaction of doses × strains: *F*_2,42_ = 1.77, *P* > 0.05 in the striatum and *F*_2,42_ = 1.28, *P* > 0.05 in the SNpc). Prodynorphin deficiency also potentiated the MA-elicited DAergic neurodegeneration as compared with Dyn^+/+^, although compared with MPTP, MA induced less damage on nigral TH-positive neurons. These results suggested that the loss of endogenous prodynorphin increases the vulnerability of DAergic neurons to MPTP or MA insult.

To determine the differences in biochemical changes of DAergic neurons in both Dyn^+/+^ and Dyn^−/−^ mice in response to neurotoxins, we examined TH expression and DA and its metabolites (DOPAC and HVA) in the striatum 7 days after final MPTP or MA injection. As shown in Figure 3, DA, DOPAC, and HVA levels were significantly decreased in both strains of mice after MPTP treatment (main effect of doses: *F*_2,42_ = 73.8, *P* < 0.001 for TH; *F*_2,42_ = 80.2, *P* < 0.001 for DA; *F*_2,42_ = 37.8, *P* < 0.001 for DOPAC; and *F*_2,42_ = 8.76, *P* < 0.001 for HVA) or MA (main effect of doses: *F*_2,42_ = 26.0, *P* < 0.001 for TH; *F*_2,42_ = 44.7, *P* < 0.001 for DA; *F*_2,42_ = 1.64, *P* > 0.05 for DOPAC; and *F*_2,42_ = 3.82, *P* < 0.05 for HVA). Consistent with the loss of TH-positive neurons after treatment with toxins (Figure 3), TH expression (Figure 3A and B) and DA (Figure 3C and D) showed more pronounced reductions after treatment with MPTP (main effect of strains: *F*_1,42_ = 10.3, *P* < 0.01 for TH; *F*_1,42_ = 8.02, *P* < 0.001 for DA; *F*_1,42_ = 0.377, *P* > 0.05 for DOPAC; and *F*_1,42_ = 1.00, *P* > 0.05 for HVA) or MA (main effect of strains: *F*_1,42_ = 5.35, *P* < 0.05 for TH; *F*_1,42_ = 11.6, *P* < 0.01 for DA; *F*_1,42_ = 1.51, *P* > 0.05 for DOPAC; and *F*_1,42_ = 1.30, *P* > 0.05 for HVA) in Dyn^−/−^ mice than in Dyn^+/+^mice. The greater depletion of striatal DA in Dyn^−/−^ mice was accompanied by a higher DA turnover rate, as expressed by the ratio of metabolites and DA (main effect of doses: *F*_2,42_ = 23.0, *P* < 0.001 for MPTP; *F*_2,42_ = 19.8, *P* < 0.001 for MA; main effect of strains: *F*_1,42_ = 13.2, *P* < 0.001 for MPTP; *F*_1,42_ = 5.22, and *P* < 0.05 for MA) (Figure 3I and J). A significant interaction between doses and strains was observed in several parameters after MPTP treatment (*F*_2,42_ = 3.94, *P* < 0.05 for TH; *F*_2,42_ = 2.61, *P* > 0.05 for DA; *F*_2,42_ = 1.08, *P* > 0.05 for DOPAC; *F*_2,42_ = 0.06, *P* > 0.05 for HVA; and *F*_2,42_ = 7.03, *P* < 0.01 for the DA turnover rate) or MA (*F*_2,42_ = 0.487, *P* > 0.05 for TH; *F*_2,42_ = 0.420, *P* > 0.05 for DA; *F*_2,42_ = 1.88, *P* > 0.05 for DOPAC; *F*_2,42_ = 0.02, *P* > 0.05 for HVA; and *F*_2,42_ = 3.97, *P* < 0.05 for the DA turnover rate). The higher degree of DA depletion in Dyn^−/−^ mice was consistent with more severe behavioral impairment (Figure 1) elicited by both MPTP and MA.

### Prodynorphin deficiency increases MPTP- and MA-elicited microglial activation

We reported previously that microglial activation followed by neuronal damage (reactive microgliosis) plays an active role in the progressive nature of PD, which further exacerbates DAergic neuronal loss, fueling a self-renewing cycle [42-44]. To define the role of endogenous dynorphin in microglial activation induced by MPTP or MA, we compared the time-dependent changes in microglial morphology in the nigrostriatal area between Dyn^+/+^ and Dyn^−/−^ mice (Figure 4). Because the statistical differences between the two strains in behavioral deficits and dopaminergic impairments were more pronounced at the higher dose of MPTP or MA (Figures 1,2, and 3), we selected the dose of 20 mg/kg MPTP or 7 mg/kg MA for evaluating time-dependent changes in microglial activation (Figure 4) and M1/M2 microglial differentiation (Figure 5) in Dyn^+/+^ and Dyn^−/−^ mice. Activation of microglia was morphologically observed by Iba1 immunostaining at 1, 3, and 7 days after the final injection of MPTP or MA. Although resident microglia from both Dyn^+/+^ and Dyn^−/−^ show a basal level of Iba1 expression, they appeared small and bore thin processes (Figure 4). One day after the final injection of MPTP or MA, activated microglia characterized by intensified Iba1 staining and enlarged cell size, were distributed throughout the Dyn^+/+^, which continued for 7 days (Figure 4A and B). Quantitative analysis of Iba1 staining in the striatum (main effect of time points: *F*_3,56_ = 24.8, *P* < 0.001 for MPTP; *F*_3,56_ = 15.6, *P* < 0.001 for MA) and SN (main effect of time points: *F*_3,56_ = 19.4, *P* < 0.001 for MPTP; *F*_3,56_ = 12.9, *P* < 0.001 for MA) demonstrated a marked increase after neurotoxin treatment (Figure 4C-F). Interestingly, more pronounced microglial activation elicited by MPTP or MA was observed in both the striatum (main effect of strains: *F*_1,56_ = 20.3, *P* < 0.001 for MPTP; *F*_1,56_ = 6.03, *P* < 0.05 for MA) and SN (main effect of strains: *F*_1,56_ = 14.3, *P* < 0.001 for MPTP; *F*_1,56_ = 4.51, and *P* < 0.05 for MA) of Dyn^−/−^ mice, characterized by a greater increase in Iba1 staining intensity and enlarged cell size compared with that in Dyn^+/+^ mice. However, a significant interaction between time points and strains was not induced by MPTP (interaction of time points × strains: *F*_3,56_ = 1.60, *P* > 0.05 in the striatum; *F*_3,56_ = 1.43, *P* > 0.05 in the SNpc) or MA (interaction of time points × strains: *F*_3,56_ = 0.900, *P* > 0.05 in the striatum; *F*_3,56_ = 1.51, *P* > 0.05 in the SNpc). These results suggested that prodynorphin deficiency enhances microglial activation in the presence of MPTP or MA-induced neuronal damage.

### Prodynorphin deficiency promotes M1 microglial differentiation

It has been suggested that macrophages/microglia play different roles in tissue repair or damage in response to CNS injury. These divergent effects may be due to distinct macrophage/microglial subsets, that is, ‘classically activated’ proinflammatory (M1) or ‘alternatively activated’ anti-inflammatory (M2) cells. To further characterize the enhanced microglial activation after neurotoxin treatment, we measured mRNA levels of M2 (arginase 1 and CD206) (Figure 5A and C) and M1 markers (CD16, CD32, and CD86) (Figure 5E, G, and I) in the mouse striatum 1, 3, and 7 days after the final MPTP treatment. RT-PCR analysis revealed that MPTP time-dependently decreased the gene expression of both arginase 1 (main effect of time points: *F*_3,56_ = 11.1, *P* < 0.001) and CD206 (main effect of time points: *F*_3,56_ = 13.1, *P* < 0.001), two M2 phenotype markers, in both strains of mice (Figure 5A and C). Further analysis showed that the decreases in expression of these two M2 genes were more pronounced (main effect of strains: *F*_1,56_ = 9.41, *P* < 0.001 for arginase 1;*F*_1,56_ = 4.16, *P* < 0.05 for CD206) in Dyn^−/−^ mice than in Dyn^+/+^ mice (Figure 5A and C). In contrast, the levels of expression of M1 phenotype markers (that is, CD16, CD32, and CD86) were markedly increased in both strains of mice 1 day after the final MPTP treatment (main effect of time points: *F*_3,56_ = 59.1, *P* < 0.001 for CD16; *F*_3,56_ = 24.1, *P* < 0.001 for CD32; and *F*_3,56_ = 27.3, *P* < 0.001 for CD86) (Figure 5E, G, and I). Enhanced expression of M1 phenotype markers decreased gradually after 3 and 7 days; however, the expression levels of these genes were higher (main effect of strains: *F*_1,56_ = 8.18, *P* < 0.01 for CD16; *F*_1,56_ = 10.4, *P* < 0.01 for CD32; and *F*_1,56_ = 14.8, *P* < 0.001 for CD86) in Dyn^−/−^ mice than in Dyn^+/+^ mice (Figure 5E, G, and I). Prodynorphin deficiency also significantly reduced the levels of arginase 1 and CD206 gene expression in MA-treated mice compared with Dyn^+/+^ mice (main effect of time points: *F*_3,56_ = 9.46, *P* < 0.001 for arginase 1; *F*_3,56_ = 11.6, *P* < 0.001 for CD206; main effect of strains: *F*_1,56_ = 10.2, *P* < 0.01 for arginase 1; *F*_1,56_ = 7.35, *P* < 0.01 for CD206) (Figure 5B and D). However, prodynorphin deficiency significantly increased the gene expression levels of CD16 and CD86, but not that of CD32 (main effect of time points: *F*_3,56_ = 6.71, *P* < 0.001 for CD16; *F*_3,56_ = 50.1, *P* < 0.001 for CD32; and *F*_3,56_ = 18.3, *P* < 0.001 for CD86; main effect of strains: *F*_1,56_ = 12.5, *P* < 0.001 for CD16; *F*_1,56_ = 0.076, *P* > 0.05 for CD32; and *F*_1,56_ = 6.15, *P* < 0.05 for CD86) (Figure 5F-J). Particularly, CD16 gene expression showed significant interaction between time points and strains after MA treatment (*F*_3,56_ = 3.81, *P* < 0.05) (Figure 5F). However, the other parameters showed no significant interaction between time points and strains after MPTP (*F*_3,56_ = 1.40, *P* > 0.05 for arginase 1; *F*_3,56_ = 0.726, *P* > 0.05 for CD206; *F*_3,56_ = 2.18, *P* > 0.05 for CD16; *F*_3,56_ = 1.50, *P* > 0.05 for CD32; and *F*_3,56_ = 1.29, *P* > 0.05 for CD86) or MA treatment (*F*_3,56_ = 2.03, *P* > 0.05 for arginase 1; *F*_3,56_ = 0.267, *P* > 0.05 for CD206; *F*_3,56_ = 0.635, *P* > 0.05 for CD32; and *F*_3,56_ = 1.70, *P* > 0.05 for CD86). These results suggested that endogenous prodynorphin deficiency accelerates microglial differentiation to the M1 phenotype after MPTP or MA treatment.

## Discussion

Using Dyn^−/−^, we demonstrated that endogenous prodynorphin deficiency aggravated behavioral impairment and DAergic neuronal loss induced by MPTP and MA. These results are consistent with our hypothesis that endogenous dynorphin plays a critical role in protecting nigrostriatal DAergic neurons from chemical insults. There were three major findings of this study: (1) MPTP- and MA-elicited impairments of locomotor activity and rota-rod performance were more pronounced in Dyn^−/−^ than in Dyn^+/+^; (2) these motor deficits were well correlated with greater nigrostriatal DAergic neuronal loss in Dyn^−/−^ than in Dyn^+/+^ after toxin treatment; and (3) both toxins triggered higher degrees of microglial activation and M1 phenotype differentiation in Dyn^−/−^ as compared with Dyn^+/+^, suggesting that overactivated microglia and amplified proinflammatory activities underlie the mechanisms of the exacerbated neurotoxic effects.

MPTP and MA are commonly used to create rodent PD models. Both toxins specifically target the nigrostriatal DA pathway. At the doses used in this study, MPTP and MA elicited both motor deficits and nigrostriatal DAergic neuronal loss. Treatment with MA is well known to result in terminal degeneration of dopaminergic neurons in the striatum [45-47]. Additionally, we [48] and others [49,50] have demonstrated that acute toxic dosing of MA can induce nigral degeneration, which is thought to parallel the pathological changes observed in the Parkinsonian condition [48,49,51]. However, whether MA-induced nigral degeneration is due to retrograde degeneration or direct neurotoxic effects on nigral cell bodies remains to be determined.

The overall results showed a high degree of consistency indicating that the behavioral motor deficits and DAergic neuronal losses are more pronounced in Dyn^−/−^ than in Dyn^+/+^ after neurotoxin treatment. In addition, the motor deficits were well-correlated with the impairment of nigrostriatal DAergic function as indicated by the greater loss of nigral DAergic neurons and striatal DA levels in Dyn^−/−^ as compared with Dyn^+/+^ mice (Figures 2 and 3). Taken together, the results of this study provided clear evidence that endogenous dynorphin plays a critical role in protection of nigrostriatal DAergic neurons from chemical insults.

Several studies have suggested that MA-induced hyperthermia mediates its neurotoxicity [52,53]. However, other reports have indicated that hyperthermia per se may not fully explain the MA-induced neurotoxicity, and that other factors may be involved in its neurotoxic effects [54-56]. For instance, pretreatment with reserpine, a drug known to produce hypothermia, did not prevent MA-induced neurotoxicity [54,55]. In the present study, we failed to obtain any significant difference in MA-induced hyperthermic response between Dyn^−/−^ and Dyn^+/+^ mice (see Additional file 1 as Additional file 2: Figure S2), suggesting that the dynorphin-mediated neuroprotective mechanism in response to MA toxicity may not require thermal regulation.

The possible mechanism underlying the neuroprotective effect of endogenous dynorphin was also examined in this study. Based on the results of our previous cell culture studies, we hypothesized that endogenous dynorphin-mediated neuroprotection is mediated mainly via its potent anti-inflammatory properties. In this *in vivo* study, we examined the activation and phenotype shifting of microglia after MPTP or MA treatment. Although both MPTP and MA are known to directly damage DAergic neurons, our previous *in vitro* observations suggested that dynorphin-mediated neuroprotection is not due to its direct protective effect on neurons. Instead, there is evidence that dynorphin shows its potent anti-inflammatory properties by inhibiting overactivation of microglia through a process called reactive microgliosis, which occurs when neurons are damaged by neurotoxins or other insults. Microgliosis has been traditionally considered to serve a passive role in the removal of dead or damaged neurons and debris by phagocytosis. However, it is now clear that microglial cells are reactivated during microgliosis, and further exacerbate neurodegeneration under severe inflammatory conditions. We have shown that many noxious endogenous compounds appear in the extracellular milieu following neuronal injury, and are capable of causing activation of microglia leading to reactive microgliosis. These compounds include membrane breakdown products, abnormally processed, modified, or aggregated proteins (for example, α-synuclein and β-amyloid) [57], and leaked cytosolic compounds (for example, α-synuclein, neuromelanin, μ-calpain, and high-mobility group box 1) [44,58,59].

Activation of the MAC-1/PHOX axis and subsequent production of superoxide underlie the potential mechanisms. Our recent studies have provided convincing evidence indicating that ’reactive microgliosis’ plays a critical and active role in the formation of a self-propelling vicious cycle and drives the progression of neurodegeneration [12,13,42]. The importance of reactive microgliosis in the progression of PD has been characterized in MPTP and MA models of PD [60-62]. Thus, we believe the neuroprotective role of dynorphin is mainly mediated through its anti-inflammatory effect in slowing down this vicious cycle. Consistent with changes in motor deficits and loss of nigrostriatal DAergic neurons, both toxins elicited more pronounced activation of microglia in the nigrostriatal area based on both the number of activated microglia and Iba1 immunoreactivity (Figure 4).

To further characterize the phenotype of microglia, we measured the expression of several markers indicative of M1 and M2 microglia. As mentioned above, microglia in the brain have the classically activated M1 phenotype or alternatively activated M2 phenotype depending on the inflammatory conditions of the local microenvironment. Both MPTP and MA treatment enhanced the mRNA level expression of M1 markers (CD16, CD32, and CD86), while those of M2 markers (arginase 1 and CD206) were decreased (Figure 5). These results indicated that microglia after toxin treatment existed mainly in the classical activation state, which is highly proinflammatory. Endogenous dynorphin may serve to dampen this neuroinflammatory process. The detailed anti-inflammatory effects of dynorphin were investigated in our previous cell culture studies [12,14], and the results indicated that inhibition of microglial NADPH oxidase-generated superoxide production is the main site of action of dynorphin. Decreased superoxide production by this peptide reduces the release of various proinflammatory factors, including cytokines and prostaglandins, and dampens inflammation [12,14,63]. Taken together, the findings of this *in vivo* study are consistent with the hypothesis that endogenous dynorphin-elicited neuroprotection is mainly mediated through its potent anti-inflammatory properties.

It is worth noting that there was an interesting reciprocal interaction between dynorphin and substance P, which are colocalized in the striatonigral pathway, in their regulation of microglial activation and subsequent DAergic neuron survival. In contrast to dynorphin, our previous study showed that subpicomolar concentrations of substance P produced microglia-dependent DAergic toxicity in midbrain neuron-glia cultures [13]. Our preliminary data indicated that MPTP treatment produced less neuroinflammation and loss of DAergic neurons in the nigrostriatal regions in substance P-deficient mice (Wang *et al*., unpublished data), indicating converse roles of these two peptides in regulating DAergic neuron survival. At present, the homeostatic mechanisms regulating microglial function in the SN are unknown. Based on the present *in vivo* findings plus the observations that dynorphins and substance P can affect nigral DAergic neuron survival at subpicomolar concentrations in cell cultures [12,13], it is interesting to speculate that the balance of these two endogenous peptides appears to be critical for microglial activation and long-term survival of nigrostriatal DAergic neurons. We hypothesize that while peptide modulation of acute DA neuronal activity occurs in the synapse at micromolar concentrations, the physiologically relevant concentrations required to regulate microglial activity are much lower (possibly of the subpicomolar order), occurring after the peptides diffuse from the synapse to outside the junction. We further hypothesize that the sites of action of subpicomolar-acting peptides are the microglial cells, which are outside the synaptic junction (presumably the concentrations of peptides must be much lower than those in the synaptic junction), and are associated with long-term regulation of neuronal survival.

## Conclusions

In summary, this is the first report indicating that endogenous dynorphin deficiency exacerbates motor deficits and DAergic neuronal losses in MPTP- and MA-induced rodent PD models through amplified microglial activation. These results support the hypothesis that endogenous dynorphin serves as a protector against inflammatory damage in the progression of PD. This study and our ongoing research on substance P will reveal important novel functions of these two peptides in long-term regulation of nigrostriatal DAergic neuron survival and their roles in the pathogenesis of PD.

## Abbreviations

6-OHDA, 6-hydroxydopamine; CNS, central nervous system; CRE, cAMP response elements; DA, dopamine; Dyn, dynorphin; L-dopa, levodopa; LPS, lipopolysaccharide; MA, methamphetamine; MPTP, 1-methyl-4-phenyl-1,2,3,6-tetrahydropyridine; PD, Parkinson’s disease; RT-PCR, reverse transcription and polymerase chain reaction; SN, substantia nigra; ST, striatum.

## Misc

Qingshan Wang and Eun-Joo Shin are contributed equally

## Competing interests

The authors declare they have no actual or potential competing financial interests.

## Authors’ contributions

EJS and XKTN carried out the behavioral experiments. XKTN carried out RT-PCR and Western blot analyses. EJS performed immunocytochemistry, and participated in the data interpretation. QW and HCK drafted the manuscript. QL and JHB helped to carry out the behavioral experiments and RT-PCR. HCK and JSH conceived the study, participated in its design and coordination. EJS, WKK, and GB helped to draft the manuscript. All of the authors have read and approved the final manuscript.

## Supplementary Material

Additional file 1 Supplemental Informations. Click here for file

Additional file 2 **Figure S1. **Changes in locomotor activity (A) and rota-rod performance (B) induced by MPTP or MA in Dyn^**+/+**^ mice. Each value represents the mean ± standard error of the mean (S.E.M.) of 10 mice. *P <0.05, **P <0.01, ***P <0.001 vs. the respective control saline group, &*P* <0.05, &&*P* <0.01 vs. respective control groups 3 days after the final treatment, §*P* <0.05, §§*P* <0.01 vs. respective control groups 7 days after the final treatment (two-way repeated-measures ANOVA followed by *post-hoc* Tukey’s HSD test or pairwise comparison with paired *t*-test). **Figure S2.** Prodynorphin gene deficiency did not affect hyperthermia induced by MA. Dyn^+/+^, prodynorphin gene wild-type mice; Dyn^−/−^, prodynorphin gene knockout mice. Dyn^+/+^ and Dyn^−/−^ received four MA injections (5 or 7 mg/kg, intraperinoteally) at 2-h intervals. Rectal temperature was measured 1 h after each MA injection. Ambient temperature: 21 ± 1°C. Each value is the mean ± S.E.M of eight mice. *P <0.001 vs. the respective control saline group (three-way repeated-measures ANOVA followed by *post-hoc* Tukey’s HSD test or pairwise comparison with paired *t*-test). Click here for file
