# Supplementary material for: Endogenous dynorphin protects against neurotoxin-elicited nigrostriatal dopaminergic neuron damage and motor deficits in mice
Source: J Neuroinflammation. 2012 Jun 13;9:124. doi: 10.1186/1742-2094-9-124 (PMC3409049; doi:10.1186/1742-2094-9-124)
Supplement: Additional file 1 — Supplemental Informations. [file 1742-2094-9-124-S1.doc]

**Supplemental Informations**

**Supplemental Methods**

**Animals and drug treatments**

To understand whether mice show spontaneous behavioral recovery after repeated treatment with MPTP or MA, we employed 6-month-old Dyn^+/+^ mice. For more details, see the Methods section in the text.

**Measure of rectal temperature**

Rectal temperature (under ambient temperature: 21 ± 1°C) was measured at 1 h after each MA injection to examine whether prodynorphin gene deficiency affects MA-induced hyperthermia. Measurement was done by inserting a thermometer probe lubricated with oil at least 3 cm into the rectum of the mice. To prevent sudden movements, especially in MA-treated mice, animals were gently handled with a wool glove while their tail was moved to allow probe insertion. This was done to reduce any effect of restraint stress on rectal temperature. When the attempt to insert probe was not successful (i.e., sudden movements of the animal or the need to restrain the mouse), the animal was excluded from the group [23].

**Statistics**

Data were analyzed by two-way repeated-measures ANOVA, with doses as the between-subjects factor and time as the within-subjects factor (Supplemental Figure 1), or three-way repeated-measures ANOVA, with doses and strains as between-subjects factors and time as a within-subjects factor (Supplemental Figure 2). When the main effect of strains, doses, or time was significant, *post-hoc* Tukey’s HSD test (among doses for each time in the same strain) or pairwise comparison using paired *t*-test (among measurements for each dose or between two strains for each dose at each measurement) was performed. A *p-*value less than 0.05 was deemed to be statistically significant.

**Supplemental Results**

**Behavioral impairments induced by MPTP or MA lasted for at least 7 days after the final treatment in Dyn^+/+^ mice.**

As shown in Supplemental Figure 1, either dopaminergic neurotoxin-induced reduction in locomotor activity was evident in a dose- and time-dependent manner [dose (main effect of doses: *F*_2,27_ = 47.3, *P* < 0.001 for MPTP; *F*_2,27_ = 13.2, *P* < 0.001 for MA) and time (main effect of time points: *F*_2,54_ = 11.5, *P* < 0.001 for MPTP; *F*_2,54_ = 3.33, *P* < 0.005 for MA; interaction of doses × time points: *F*_4,54_ = 4.41, *P* < 0.01 for MPTP; *F*_4,54_ = 0.905, *P* > 0.05 for MA)].

Pairwise comparison using paired *t*-test revealed significant recovery in locomotor activity in mice receiving MPTP (15 and 20 mg/kg: 3 d or 7 d vs. 14 d, *P* < 0.01 or *P* < 0.01, respectively) or MA (5 mg/kg: 3 d or 7 d vs. 14 d, *P* < 0.05 or *P* < 0.05, respectively) at 14 days after the final injection (Supplemental Figure 1A and B). Changes in rota-rod performance were consistent with those in locomotor activity (main effect doses: *F*_2,27_ = 32.0, *P* < 0.001 for MPTP; *F*_2,27_ = 21.8, *P* < 0.001 for MA; main effect of time points: *F*_2,54_ = 5.44, *P* < 0.01 for MPTP; *F*_2,54_ = 2.07, *P* > 0.05 for MA; interaction of doses × time points: *F*_4,54_ = 2.07, *P* > 0.05 for MPTP; *F*_4,54_ = 0.610, *P* > 0.05 for MA). Pairwise comparison using a paired *t*-test revealed that rota-rod performance was also significantly recovered at 14 days after the final treatment with MPTP (15 mg/kg: 3 d or 7 d vs. 14 d, *P* < 0.05 or *P* < 0.05, respectively; 20 mg/kg: 3 d vs. 14 d, *P* < 0.05) (Supplemental Figure 1C and D). These results suggest that spontaneous behavioral recovery did not occur, at least within 7 days, under our experimental condition.

**Prodynorphin deficiency did not affect MA-induced hyperthermia.**

Significant hyperthermia was induced by MA injection in a dose-dependent manner; however, no significant difference was found between the two strains (main effect doses: *F*_2,42_ = 162, *P* < 0.001; main effect of strains: *F*_1,42_ = 1.16, *P* > 0.05; main effect of time: *F*_4,168_ = 35.9, *P* < 0.001; interaction of doses × strains: *F*_2,42_ = 0.246, *P* > 0.05; interaction of doses × time: *F*_8,168_ = 21.4, *P* < 0.001; interaction of strains × time: *F*_4,168_ = 2.34, *P* > 0.05; interaction of doses × strains × time: *F*_8,168_ = 0.874, *P* > 0.05) (Supplemental Figure 2). These results suggest that dynorphin-mediated neuroprotective effect in response to MA toxicity may not require a thermoregulatory mechanism.

**Supplemental Figure Legends**

**Supplemental Figure 1. Changes in locomotor activity (A) and rota-rod performance (B) induced by MPTP or MA in Dyn^+/+^ mice.** Each value represents the mean ± standard error of the mean (S.E.M.) of 10 mice. **p* <0.05, ***p* <0.01, ****p* <0.001 vs. the respective control saline group, &*p* <0.05, &&*p* <0.01 vs. respective control groups 3 d after the final treatment, §*p* <0.05, §§*p* <0.01 vs. respective control groups 7 d after the final treatment (two-way repeated-measures ANOVA followed by *post-hoc* Tukey’s HSD test or pairwise comparison with paired *t*-test ).

**Supplemental Figure 2. Prodynorphin gene deficiency did not affect hyperthermia induced by MA.** Dyn^+/+^, prodynorphin gene wild-type mice; Dyn^-/-^, prodynorphin gene knockout mice. Dyn^+/+^ and Dyn^-/-^ received four MA injections (5 or 7 mg/kg, intraperinoteally) at 2-h intervals. Rectal temperature was measured 1 h after each MA injection. Ambient temperature: 21 ± 1°C. Each value is the mean ± S.E.M of eight mice. **p* <0.001 vs. the respective control saline group (three-way repeated-measures ANOVA followed by *post-hoc* Tukey’s HSD test or pairwise comparison with paired *t*-test).
