# Supplementary material for: Endogenous dynorphin protects against neurotoxin-elicited nigrostriatal dopaminergic neuron damage and motor deficits in mice
Source: J Neuroinflammation. 2012 Jun 13;9:124. doi: 10.1186/1742-2094-9-124 (PMC3409049; doi:10.1186/1742-2094-9-124)
Supplement: Additional file 2 — Figure S1. Changes in locomotor activity (A) and rota-rod performance (B) induced by MPTP or MA in Dyn+/+ mice. Each value represents the mean ± standard error of the mean (S.E.M.) of 10 mice. *P <0.05, **P <0.01, ***P <0.001 vs. the respective control saline group, &P <0.05, &&P <0.01 vs. respective control groups 3 days after the final treatment, §P <0.05, §§P <0.01 vs. respective control groups 7 days after the final treatment (two-way repeated-measures ANOVA followed by post-hoc Tukey’s HSD test or pairwise comparison with paired t-test). Figure S2. Prodynorphin gene deficiency did not affect hyperthermia induced by MA. Dyn+/+, prodynorphin gene wild-type mice; Dyn−/−, prodynorphin gene knockout mice. Dyn+/+ and Dyn−/− received four MA injections (5 or 7 mg/kg, intraperinoteally) at 2-h intervals. Rectal temperature was measured 1 h after each MA injection. Ambient temperature: 21 ± 1°C. Each value is the mean ± S.E.M of eight mice. *P <0.001 vs. the respective control saline group (three-way repeated-measures ANOVA followed by post-hoc Tukey’s HSD test or pairwise comparison with paired t-test). [file 1742-2094-9-124-S2.ppt]

## Slide 1
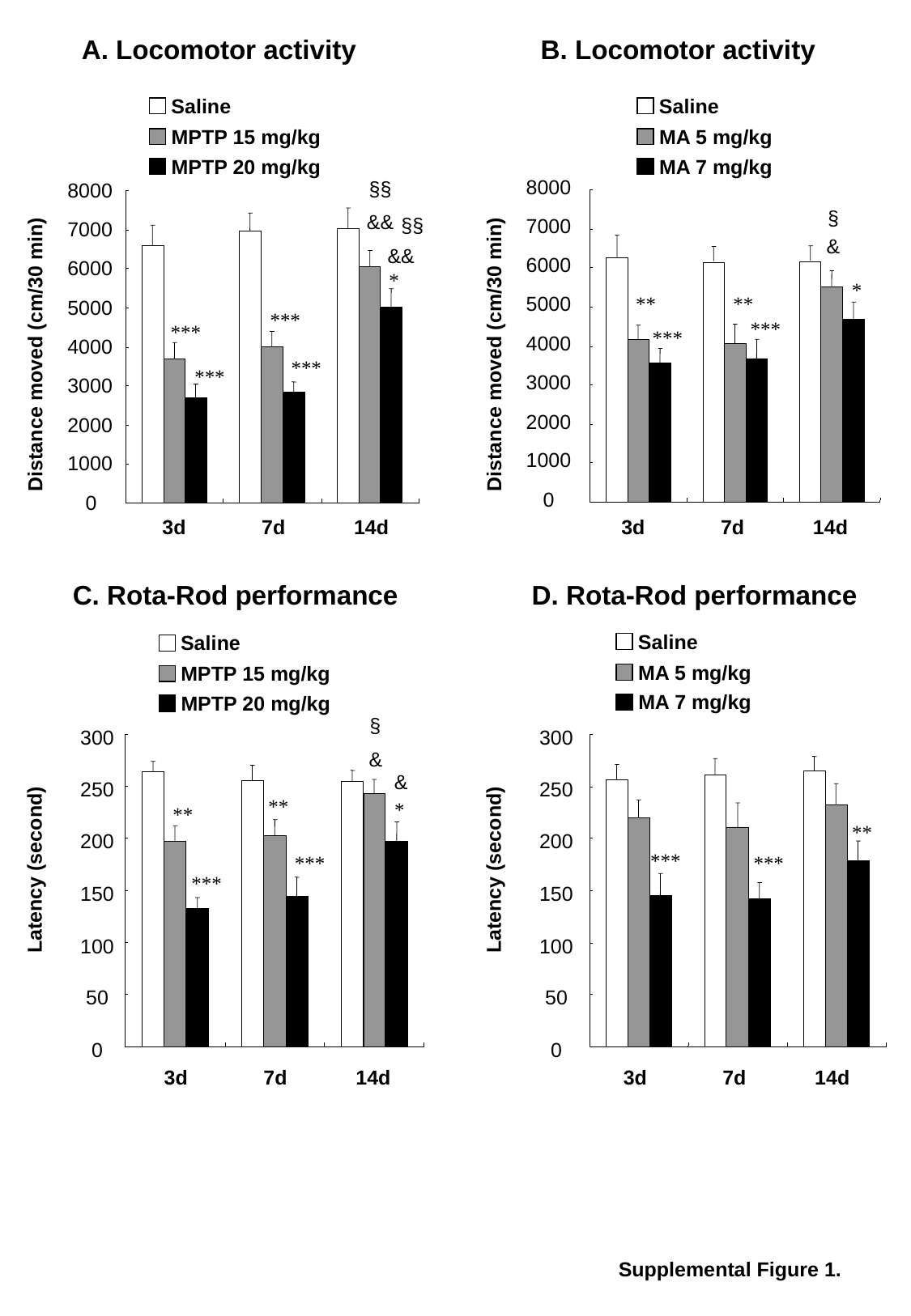

A. Locomotor activity
B. Locomotor activity
Saline
Saline
MPTP 15 mg/kg
MA 5 mg/kg
MPTP 20 mg/kg
MA 7 mg/kg
§§
8000
8000
§
&&
§§
7000
7000
&
&&
6000
6000
*
*
**
**
5000
5000
***
***
***
***
4000
4000
Distance moved (cm/30 min)
Distance moved (cm/30 min)
***
***
3000
3000
2000
2000
1000
1000
0
0
3d
7d
3d
7d
14d
14d
C. Rota-Rod performance
D. Rota-Rod performance
Saline
Saline
MA 5 mg/kg
MPTP 15 mg/kg
MA 7 mg/kg
MPTP 20 mg/kg
§
300
300
&
&
250
250
**
*
**
**
200
200
***
***
***
Latency (second)
Latency (second)
***
150
150
100
100
50
50
0
0
3d
7d
3d
7d
14d
14d
Supplemental Figure 1.

## Slide 2
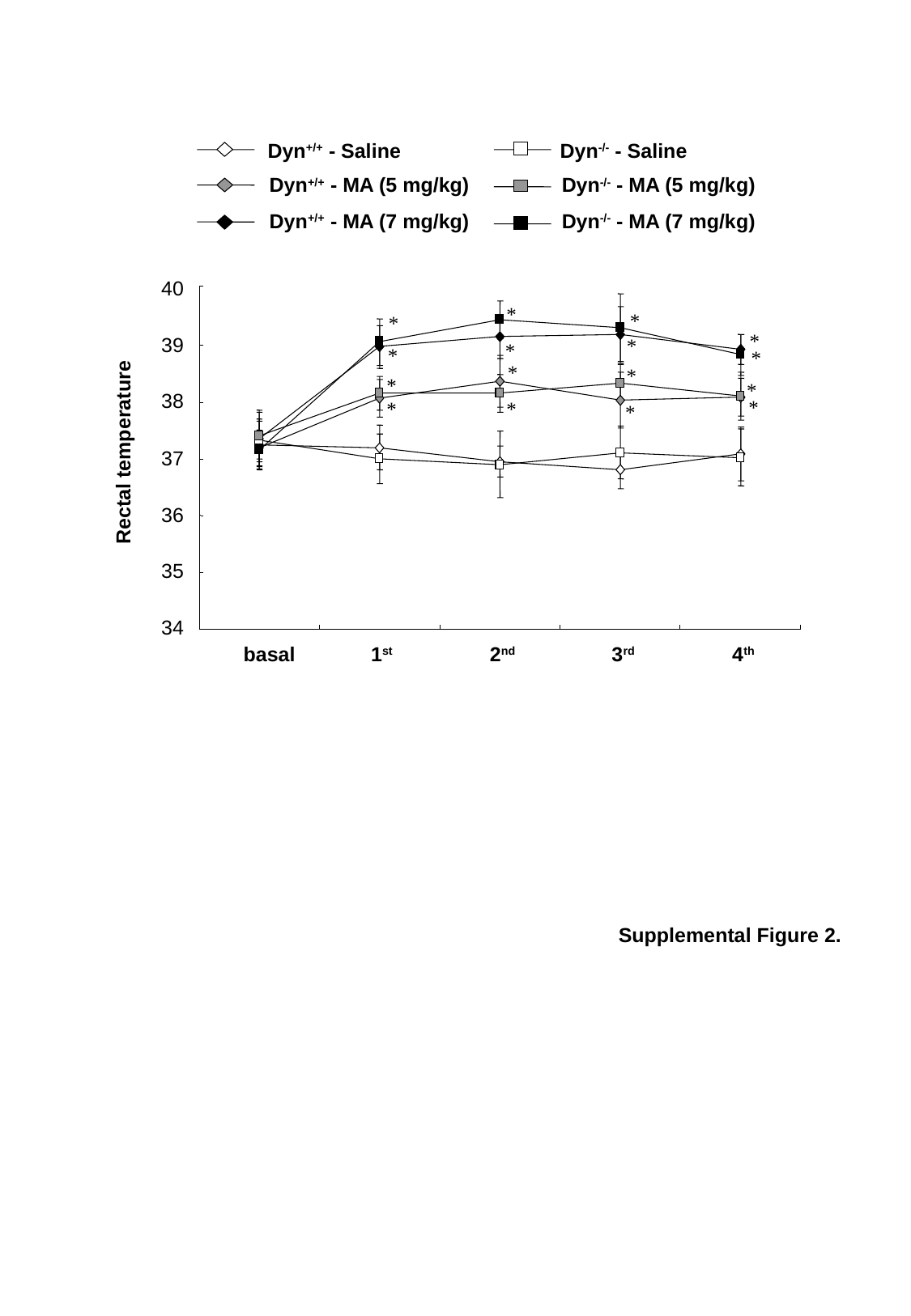

Dyn+/+ - Saline
Dyn-/- - Saline
Dyn+/+ - MA (5 mg/kg)
Dyn-/- - MA (5 mg/kg)
Dyn+/+ - MA (7 mg/kg)
Dyn-/- - MA (7 mg/kg)
40
*
*
*
*
*
*
39
*
*
*
*
*
*
38
*
*
*
*
Rectal temperature
37
36
35
34
basal
1st
2nd
3rd
4th
Supplemental Figure 2.
